# Whither the University? Universities of Technology and the Problem of Institutional Purpose

**DOI:** 10.1007/s11948-019-00147-7

**Published:** 2019-12-23

**Authors:** Seumas Miller

**Affiliations:** 1 Charles Sturt University, Canberra, Australia; 2 TU Delft, Delft, The Netherlands; 3 University of Oxford, Oxford, UK

**Keywords:** University, University of technology, Epistemic institutions, Joint epistemic action, Knowledge for its own sake, Market-based institutions, Bureaucratisation, Marketisation, John Henry Newman

## Abstract

There is a need to provide an appropriate normative conception of the modern university: a conception which identifies its unifying purposes and values and, thereby, gives direction to institutional role occupants, governments, public policymakers and other would-be institutional designers. Such a conception could admit differences between modern universities; differences, for example, between so-called universities of technology and other universities. Indeed, it is preferable to frame the issue at the level of higher education or university systems rather than at the level of individual universities. According to the teleological normative theory of social institutions, social institutions are organizations or systems of organizations that provide collective goods by means of joint activity; universities are no exception. So what are the fundamental collective good(s) that universities of technology, or the larger systems of which they are a part, ought to be providing and how are they travelling in this regard? This is the question addressed in this paper.

## Introduction: The Modern University

The idea or, more formally, the informing normative conception, of a university, as with any human institution, is an evolving one. Mediaeval universities, such as Oxford or Bologna, are very different places from contemporary ones such as Delft University of Technology in the Netherlands, Charles Sturt University in Australia or Beijing University in China or, for that matter, Harvard University in the United States or, indeed, Oxford or Bologna universities today. Moreover, different universities need to take on somewhat different forms in different historical and socio-economic contexts. Oxford used to have as a primary institutional aim to provide education for the clergy, and prior to 1920 admitted only male students. Today many universities, such as Charles Sturt, provide an education for new and emerging professions, including ones dominated by women, e.g., nurses and social workers, and other universities, such as Delft prioritize teaching and research in specific fields of science and technology.

As with any institution, any given university—indeed universities in general—can either flourish or decay. A hundred years ago who would have confidently claimed that in some Western countries a majority of those who leave school, whether or not they graduate, would go on to attend a university and that it would be widely held that modern economies require that this be so. Further, universities—like other institutions—can serve a variety of interests, some legitimate, some not. For example, for many years some universities in South Africa excluded black South Africans, and developed and disseminated the ideology of apartheid.

Notwithstanding the important differences between universities, there is a need, as there is with any institution, to provide an appropriate normative conception of the university and, specifically, the modern university: a conception which identifies its unifying purposes and values and, thereby, gives direction to institutional role occupants, governments, public policymakers and other would-be institutional designers. Naturally, such a conception could admit differences between modern universities; differences, for example, between so-called universities of technology and other universities. Indeed, as I suggest below, it might be preferable to frame the issue at the level of higher education or university systems rather than at the level of individual universities. If so, the required normative conception is for a university system as a whole, e.g., that of the Netherlands, albeit there would still be a need for normative conceptions of individual parts of the system, i.e., for individual universities, and for the role of individual disciplines at the system wide and at the individual institution levels.

Unfortunately, when it comes to the modern university (or system of universities—for the sake of simplicity in this paper I often use the term “university” to refer to both single institutions and systems thereof, and disambiguate only where necessary—there is no such agreed normative conception on offer. Rather there is an unresolved theoretical or intellectual problem concerning the very nature and role of the university as an institution. It is now, I suggest, quite unclear what the specific purposes of the modern university are supposed to be. To some it is self-evident that its goals must be fundamentally economic, e.g., to contribute to the nation’s wealth; to others that its goals must be social, e.g., to bring about a society of equals. Still others argue that it ought to have the more traditional role of acquiring knowledge for its own sake but offer no account of what the knowledge in question is, e.g., which specific areas of knowledge ought to be taught/researched and on what grounds. The widespread acceptance of such simplistic views as these serves only to point to the absence of an acceptable worked-out conception of the role of the university; a conception which would doubtless embrace the pursuit of knowledge for its own sake as well as the realization of wider social and economic goals, but do so in a manner that not only provided further specification of these general goals but also integrated them with a view to resolving the inevitable conflicts between them. Nor am I alone in drawing attention to this lacuna. The influential British educational theorist Ronald Barnett has this to say about modern university systems: “Put simply, we have no modern educational theory of higher education” (Barnett 1990, p. 4). In his more recent work Barnett makes essentially the same point (Barnett 2013). He argues that all the existing conceptions or theories of the university are inherently problematic and, therefore, there is a need for a new conception. He does advocate a procedure which in general terms consists in taking these existing conceptions as the starting point and ‘imagining’ more adequate alternatives. However, the problem remains.

The absence of an acceptable normative theory has to some extent been masked by the process of institutional evolution that has actually been taking place. This evolution is, I suggest, a threefold process comprised of: (1) a shift from elite institutions to institutions of mass higher education (the so-called ‘massification of higher education’); (2) a process of market-driven ‘corporatization’ [so-called ‘academic capitalism’ (Slaughter and Leslie 1997)], and; (3) a process of ‘bureaucratization’ [to some extent in the name of managing and assuring quality (Brennan and Tahla 2000)]. The endpoint of this evolutionary process is unclear—and no doubt differs somewhat from one university to another, one higher education system to another—but the result thus far is that many universities in the US, the UK, Europe and Australasia are now hybrid institutions (Miller 2000). On the one hand, traditional academic values and purposes, such as the academic autonomy of individual academics and their ownership of the intellectual property they create, collegial decision-making structures, the maintenance of scholarly virtues, and the pursuit of knowledge for its own sake continue to be acknowledged or, at least, paid lip-service. On the other hand, many universities have in fact become preoccupied with, even dominated by, essentially bureaucratic, and ‘factory’ and market-based values and purposes.

In pointing to these processes of massification, marketization and bureaucratization one is not denying that universities have always, and necessarily, had an administrative arm and have typically recognized the need to take cognizance of issues of supply and demand in relation to student load and funded research; much less is one ignoring the important and desirable social and economic roles that universities can have, have had, do have and should have. Rather the issue is one of degree and, ultimately, of whether these processes serve larger academic purposes; or is the tail now wagging the dog?

For convenience let us refer to this threefold process of massification, marketization and bureaucratization overlaid on traditional (for want of a better term) universities as hybridization. Hybridization is evident structurally and culturally, and in respect of the preponderance and purposes of institutional activities. Consider structure. The traditional collegial structure is characterized by an emphasis on individual academic autonomy in the context of leadership provided by an academic professoriate—the so-called ‘community of scholars’. This governance structure has largely been replaced, or at least overlaid, by a top-down hierarchical structure of academic managers deploying various measurement devices, e.g., numbers of publications, success in research grant applications and student teaching evaluations, as management tools in the service of enhancing performance.

Now consider institutional activity. Here there has been a substantial shift from academic activity in favor of what are essentially bureaucratic and market-based activities. Indeed, at many, if not most, modern universities, academics are now outnumbered by non-academic staff. And, as for academics, they spend much less of their time on the purely academic activities of teaching and research and far more of their time on administrative and market activities. Moreover, their teaching is frequently undertaken in the context of very large classes and declining staff/student ratios, and their research, especially research in science and technology, undertaken for government or private sector agencies and funded by these external agencies.

Naturally, in many cases, especially in the humanities and social sciences, government funding decisions, in particular, are mediated by academic quality assurance processes and, indeed, pertain to research projects initiated by academics. However, even in these cases governments provide ‘research directions’, albeit at arms-length.

It is surely illusory to believe that academic standards and scholarly teaching virtues have been maintained in the context of mass higher education; unless one believes in miracles a substantial increase in input (student numbers) combined with an under-resourced process (declining staff/student ratios) will surely lead to a decline in the quality of the output (the intellectual quality of graduating students).

It is also naïve to believe that, for example, the carrot (if not the stick) of large-scale government and private sector research grants and consultancies, university technology transfer/external partnership arrangements, university-owned intellectual property, etc. has not significantly impacted on academics’ commitment to pursuing research for its epistemic, as opposed to (say) its commercial, value. In fact David Resnik has offered detailed arguments to the conclusion that it has so impacted, at least in the US university R&D sector (Resnik 2007). According to Resnik (2007, p. 5), 10% of R&D in US universities is sponsored by private industry. Moreover, he claims that scientists are typically hired in large part because of their ability to attract government grants (Resnik 2007, p. 13). The argument that Resnik makes is obviously especially relevant to those universities with a dominant focus on science and technology, notably, universities of technology.

This is not to deny the benefits that have accrued from, for example, external funding; nor is it to believe in some past ‘golden age’ in which all was well with the academy. Rather the issue is (again) one of emphasis; has not the pendulum swung too far in the direction of externally driven research at the expense, in particular, of epistemically significant research? And if not, by the lights of what normative account is this to be concluded?

Predictably, there have also been important cultural changes. Let us take culture to be the ethos or ‘spirit’ which informs an organization, occupation or social group. Being largely a matter of, so to speak, collective attitudes—attitudes embodied in informal narratives, rituals, and the like—culture may or may not coincide with actual processes, practices and purposes. Nevertheless, at the very least culture in this sense determines the manner in which organizational activity is undertaken. Formerly, university culture was such that it privileged academics and academic activity above all else; the heart and soul of the university was felt to be the community of autonomous scholars whose teaching and research activity was not only valued for its own sake but was regarded as the *raison d’etre* of the institution. However, this culture is at odds with the above-mentioned processes of massification, marketization and bureaucratization. The latter processes tend to spawn a culture in which academics are simply regarded as workers (so-called ‘knowledge-workers’) who are a means to an end, namely, the realization of organizational goals as determined by ‘management’ and external ‘markets’; such ‘markets’ include those determined by governments to be ‘strategically’(commercially) important, such as foreign fee-paying students or areas of research in which the nation-state in question is deemed to have a comparative economic advantage. Accordingly, managers and, indeed, academics themselves may well over time come to view academics and academic activity through this utilitarian lens. As a consequence, academics are unlikely to be seeking to embody the (arcane and sentimental?) conception of an independent thinker possessed of the virtues of objectivity, breadth of knowledge, scholarly diligence etc. and driven by a desire to acquire understanding of important matters for its own sake and, if necessary, prepared to ‘speak the truth to power’ (Said 1994), of which more below. Rather the appropriate (and emerging?) image of a modern academic is more akin to that of any employee of a large bureaucratic, market-based organization; the only difference being the nature of the tasks undertaken (epistemic rather than behavioral, so to speak) and their performance measures, e.g., student evaluations, publications in high ranking journals, research grants (as opposed to, say, quarterly sales of widgets).

It might be held that this characterization of universities as undergoing hybridization is a seriously distorted picture of what is in fact taking place, and that that it is distortion is evidenced by the manifest commitment to academic excellence on the part not only of academics but also of academic managers and, indeed, universities as organizations. Certainly academics, their managers and universities are status-driven, and this is manifest at the individual level by the at times obsequious behavior on the part of ‘ordinary’ academics—and, for that matter, academic managers—in respect of ‘academic stars’ and, at the institutional level, in mission statements, a fixation with international rankings systems, and so on. However, it might be argued that this ‘commitment’ to academic excellence is little more than an addiction to status (and associated ‘marketing hype’), both for its own sake—rather than as a warranted response to important epistemic achievements—and as a means to economic survival, if not economic flourishing, in the overall context of competitive market-based academic capitalism. Accordingly, this ‘commitment’ to academic excellence may well be quite consistent with the hybridization thesis adumbrated above.

If so, perhaps we have arrived at the nub of the problem; the problem of a theoretical normative vacuum. For this conflation of academic excellence with academic status and, in particular, the emptiness of the notion of academic excellence in play exemplifies the normative problem at hand. What is the purpose of the individual university or, perhaps more to the point, of the university system of which it is one component? Academic excellence? What is that and how is it to be achieved? As the Australian educationist Paul Bourke has put it some years ago: “It is a serious problem for [higher] education that there is now pressure for quality controls and for evaluation but no agreed statement of a system wide or institutional objectives” (Bourke 1986, p. 11). And, we might add, what are the objectives of the university of technology, in particular, and how do these objectives integrate with the objectives of the system as a whole?

Prior to constructing an acceptable normative theory of the modern university, we need a serviceable normative conception of an institution: we need, so to speak, a general theory of institutions prior to constructing our special theory of the particular institution of the university. And, of course, we need this special theory prior to offering a normative account of a particular species of university, namely, the contemporary university of technology. Here, as elsewhere in this paper, the importance of the university system as a whole needs to be kept in mind; it is this system that I suggest we need an overarching normative theory of. Naturally, in a brief paper such as this I have space only to offer a sketch of the general theory of social institutions, the special theory of the university and the (as it were) species-specific theory of universities of technology.

## A Teleological Account of Social Institutions

According to the teleological normative theory of social institutions, social institutions are organizations or systems of organizations that provide collective goods by means of joint activity (Miller 2010). The collective goods in question include the fulfillment of aggregated moral rights, such as needs-based rights for security (police organizations), material well-being (businesses operating in markets), governance (governments) and, most relevant to our purposes here, education (universities). Note that the collective goods in question include epistemic goods, such as widely shared knowledge. Moreover, the theory has a focus on both individual organizations per se, e.g., a single university, and systems of organization, e.g., the higher education sector.

The central concept in the teleological account of social institutions is that of *joint action* (Miller 1992, 2001). Here we can distinguish between joint behavioural action and joint epistemic action (Miller 2015, 2016). Behavioural action is, roughly speaking, bodily action. Epistemic action is action directed to an epistemic end or goal.

Joint actions (whether behavioural or epistemic) are actions involving a number of agents performing interdependent actions in order to realize some common goal [collective end (Miller 1992)]. Examples of joint action are: two people dancing together, a number of tradesmen building a house, and a team of scientists seeking the cure for cancer (joint epistemic action).

Joint action is to be distinguished from individual action on the one hand, and from the ‘actions’ of corporate bodies on the other. Thus an individual walking down the road, shooting at a target or making a judgment are instances of individual action. A nation declaring war or a government taking legal action against a public company are instances of *corporate* action. In so far as such corporate ‘actions’ are genuine actions involving mental states such as intentions and beliefs then they are, in my view, reducible to the individual and joint actions of human beings (Miller 2010).

As we have seen, epistemic actions are actions of acquiring knowledge. Here we can distinguish between so-called ‘knowledge-that’ and ‘knowledge-how’; the former being propositional knowledge (knowledge of the truth of some proposition), the latter being practical knowledge (knowledge of how to undertake some activity or produce some artifact).

The methods of acquiring propositional knowledge are manifold but for scientific knowledge they include observation, calculation and testimony. Moreover, the acquisition of these methods is very often the acquisition of knowledge-how, e.g., how to calculate, how to use a microscope, how to ‘read’ an x-ray chart (Miller 2018).

In the case of the engineering sciences—which typically occupy a central place in universities of technology—there is an even more obvious and intimate relationship between propositional and practical knowledge, since both are in the service of constructing or making things. Thus in order to build an airplane engineers have to have prior practical (‘how-to’) knowledge and that practical knowledge is in part comprised of propositional knowledge, e.g., with respect to load bearing capacity. Moreover, this engineering model has increasing applicability in new and emerging sciences such as synthetic biology and nanotechnology. In the case of synthetic biology, for example, scientists can develop new vaccines, enhance the virulence and transmissibility of existing pathogens, and even create new pathogens (albeit, presumably using elements of existing pathogens as building blocks).

In cases of joint epistemic action there is mutual true belief among the epistemic agents that each has the same collective epistemic end, e.g., to discover the cure for cancer. Moreover, there is typically a division of epistemic labor. Thus, in scientific cases, some scientists are engaged in devising experiments, others replicating experiments, and so on. So, as is the case with joint action more generally, joint epistemic action involves interdependence of individual action, albeit interdependence of individual epistemic action.

The further point to be made here is that there is interdependence in relation to such collective epistemic ends. This is because, given the need for replication of experiments by others, each can only know that p is the cure for cancer—to continue with our example—given that others also know this, i.e., there is interdependence in relation to the collective end of knowledge.

A collective epistemic end can be both a collective intrinsic good—and thus hopefully an end in itself—and also the means to further ends. Knowledge of the cure for cancer is a case in point. Such knowledge consists of propositional and practical knowledge; knowledge of the cure for cancer and knowledge of how to produce it. However, this knowledge has as a further (collective) end the actual production of the cure (say, a drug). And this end has in turn a still further end, namely, to save lives.

Here it is important to make a threefold distinction in respect of the pursuit of knowledge between: (1) the *disinterested* pursuit of knowledge as opposed to the pursuit of knowledge by scientists or scholars who have a special and influential interest in the outcome (that is, they are biased or otherwise have a conflict of interest), e.g., apparently researchers employed by tobacco companies had a special interest in finding that smoking did not increase the risk of cancer and this distorted their results (Resnik 2007); (2) the pursuit of knowledge *for its own sake,* e.g., a researcher for a marketing company attempting to gauge potential demand for a new brand of toothpaste might have no special and, therefore, no potentially distorting, interest in the outcome of their research (the researcher is engaged in the disinterested pursuit of knowledge) but, nevertheless, the researcher might only be undertaking the research in order to get paid rather than for the sake of the knowledge gained (the researcher is not remotely interested in patterns of demand for toothpaste per se) and; (3) the pursuit of *epistemically significant* knowledge, e.g., a researcher might be engaged in research on the development of a simple device to enable the purification of contaminated drinking water and the research may well be very important in terms of its potential for reducing water-borne diseases in poverty-stricken areas, but yet be of little significance epistemically since it contributes little to our understanding of human diseases in general or of water-borne diseases in particular.

Organizational action, including organizational action undertaken in universities, typically consists of, what elsewhere I have termed, a *layered structure of joint actions* (Miller 2001). Importantly for our purposes here there are layered structures of joint *epistemic* action (Miller 2015, 2016). Consider a crime squad, comprised of detectives, forensic scientists etc., attempting to solve a crime.

At level one, a victim, A, communicates the occurrence of the crime (say, an assault) and description of the offender to a police officer, B. But A asserting that p to B is a joint epistemic action; it is a cooperative action governed by conventions, the convention that the speaker A tells the truth and the hearer trusts the speaker to tell the truth (Miller 2016).

Also at level one, a couple of detectives interview the suspect to determine motive and opportunity; the detectives are cooperating with one another in the performance of a joint epistemic action the collective end of which is to discover motive and opportunity.

Finally, at level one, a team of forensic scientists analyze the available physical evidence e.g., the DNA of the blood samples of the offender found on the victim are matched to the suspect’s DNA; the forensic scientists are engaged in joint epistemic action to determine whether there is or is not a DNA match.

These three level one joint epistemic actions are constitutive of a level two joint epistemic action, namely, the level two joint epistemic action directed towards the collective end of determining who committed the crime. Accordingly, when each of the level one joint epistemic actions is successfully performed, then the level two joint epistemic action is successfully performed, i.e., the crime squad solves crime.

Now consider an example of a large and epistemically important scientific project conducted by a number of cooperating organizations and hundreds of scientists over many years, namely, the human genome project. The project involved multiple connected goals—collective ends—and multiple layered structures of joint action, including joint projects in publishing, undertaken to realize those goals.

In fact most organizations are hierarchical institutions comprised of task-defined roles standing in authority relations to one another, and governed by a complex network of conventions, social norms, regulations, and laws. Consider a science department in a university or the forensic laboratory in a police organization: both comprise heads of department, scientists, laboratory assistants, and so on, and the work of both is governed by scientific norms of observation, replication of experiments, etc. So most layered structures of joint action, including joint epistemic action, are undertaken in institutional settings, and scientific joint epistemic action is not an exception.

Institutions have de facto purposes/strategic directions, i.e., collective ends, such as to maximize shareholder profit (corporations), to find a cure for cancer (university research team), or to build an atomic bomb (military organization). Moreover, as we saw above, institutions also have specific structures (hierarchical, collegial, etc.) and they have specific cultures (e.g., a competitive, status-driven ethos). In this connection consider scientific activity, e.g., biological research, undertaken in three different institutional settings—that of the university, the commercial firm and the military bio-defense organization (Miller 2018). Some of the principal purposes/strategic directions (collective ends) of commercial firms, e.g., to maximize shareholder profits, are quite different from, and possibly inconsistent with, those characteristic of universities, e.g., scientific knowledge for its own sake, and quite different again from those of military research establishments, e.g., to save the lives of military personnel. Again, the hierarchical structures within a military research establishment are quite different from the more collegial structures prevailing in universities; and the structure of commercial firms is quite different again. The general point to be made here is that scientific activity is not only a form of complex joint activity (a layered structure of joint epistemic action)—it is activity that is inevitably shaped by the non-scientific institutional setting in which it is conducted, i.e., by the specific collective ends, structure and cultures of particular institutions.

Here we also need to stress the distinction between the de facto institutional collective end, structure, and/or culture from what it *ought to be*; cultures, for example, can vary greatly from one organizational setting to another, notwithstanding that the type of institution in question is the same or very similar.

In the light of the above, we can distinguish the normative account of science as a joint intellectual activity, e.g., disinterestedly pursued, epistemically significant, knowledge aimed at for its own sake such as the theory of relativity, from science as means to social or economic ends, e.g., vaccines to save lives or drugs to make money for shareholders in pharmaceutical companies. Moreover, we can distinguish both from the normative account of specific institutions in which science exists principally as a means, e.g., commercial firms (vaccines to make profit), military bio-defense organization (vaccines to save lives of our military personnel).

While the notion of an organization does not necessarily include any reference to a normative dimension, most organizations do, as a matter of contingent fact, possess a normative dimension. This normative dimension will be possessed (especially, though not exclusively) by virtue of the particular moral/immoral ends (goods) that an organization or system of organisations, e.g., a market or an higher education sector, serves, as well as by virtue of the particular moral (or immoral) activities that it undertakes.

Organizations with the above detailed normative dimension are *social institutions* (Miller 2010). So—and as already noted—institutions are often organizations, and many systems of organizations, e.g., markets, higher education systems, are also institutions.

Self-evidently, social institutions have a multifaceted ethico-normative dimension, including a moral dimension. Moral categories that are deeply implicated in social institutions include: human rights and duties, contract-based rights and obligations and, importantly I suggest, rights and duties derived from the production and ‘consumption’ of collective goods.

Collective goods of the kind I have in mind have three properties: (1) they are produced, maintained or renewed by means of the *joint activity* of members of organizations or systems of organizations, i.e., by institutional actors; (2) they are *available to the whole community* (at least in principle), and; (3) they *ought* to be produced (or maintained or renewed) and made available to the whole community since they are desirable goods and ones to which the members of the community have an (institutional) *joint moral right*.

Such goods are ones that are desirable in the sense that they ought to be desired (objectively speaking), as opposed to simply being desired; moreover, they are either intrinsic goods (good in themselves), or the means to intrinsic goods. They include, but are not restricted to, goods in respect of which there is an institutionally prior moral right, e.g., security.

Note that the scope of a community is relativized to a social institution (or set of interdependent social institutions). Roughly, a community consists in the members of an organisation those who jointly produced a collective good and/or who have a joint right to that good. In the case of the meta-institution, government, the community will consist in all those who are members of any of the social institutions that are coordinated and otherwise directed by the relevant government. So the citizens of a nation-state will count as a community on this account. But note that given the global nature of much scientific cooperation, or at least of scientific research conducted in universities, and the universal, or near universal, relevance of scientific findings and applications, the ‘communities’ in question are not necessarily, or even typically, coterminous with nation-states.

Roughly speaking, on this normative teleological account of social institutions, aggregated needs-based rights, aggregated non-needs-based human rights and other desirable goods generate collective moral responsibilities which provide the ethico-normative basis for institutions, e.g., business organizations in competitive markets, welfare institutions, police organizations, universities, etc., which fulfil those rights.

For example, the aggregate need in a community for education generates a collective moral responsibility to establish and maintain social institutions, such as schools, the members of which jointly engage in educative practices; once the relevant institutions are established, then the needy have a joint moral right, and ought to have a joint institutional right, to the education in question.

So much for the general theory of social institutions, including institutions, such as universities, the collective ends of which are collective epistemic goods. Let us now turn to the special theory of the institution of the university bearing in mind that it is the system as a whole, rather than the single organisation, which might need to be the primary focus of attention.

## Newman and Liberal Knowledge

In relation to the special theory of the university, as a first step, we can do no better than to reconsider the intellectual roots of the modern university. A comprehensive review would have to examine such writers as Humboldt, Jaspers and Ortega y Gasset, but any such reconsideration would have to take into account the views of John Henry Newman (Jaspers 1960; Newman 1947; Pelikan 1992). I focus here only on Newman. Those views were elaborated in his seminal work, *The Idea of a University* (Newman 1947). Newman’s conception remains the most influential integrated vision of the university but is now widely misunderstood. Newman’s model, defective though it is in certain respects, still has a great deal to offer qua normative theory of the university (Coady and Miller 1993). In particular, it articulates and emphasizes as central to a university’s mission, institutional goals that tend otherwise to receive merely pious lip service. Importantly, by the lights of our general theory of social institutions, these institutional goals are collective ends which are also collective goods.

Specifically, the central and defining collective good, according to Newman, is what he referred to as liberal knowledge (Coady and Miller 1993). So the key notion in Newman’s special normative theory of the university as an institution is liberal knowledge. Before turning to an elaboration of that notion I note that Newman held that universities ought to pursue knowledge and understanding for its own sake *as well as* for the social and economic good of the wider society (Coady and Miller 1993). As already noted, this proposition is surely correct; however, as also already noted, the devil is to some extent in the detail, e.g., which knowledge, which social or economic purposes? I return to this general point below.

Newman’s notion of liberal knowledge is at the heart of his conception of a university. (Here and in what follows in this section I rely heavily on Coady and Miller 1993.) This is not knowledge of the ideology of the so-called liberal side of politics, or indeed of any particular field of inquiry. Essentially, liberal knowledge is knowledge informed by human reasoning; it is not simply knowledge gained by automatic processes such as sense perception: “Knowledge is called science or philosophy when it is acted upon, informed, or if I may use a strong figure, impregnated by Reason” (Newman 1947, p. 99). Accordingly, one might surmise that the mechanical application of epistemic procedures to raw empirical data is not the kind of epistemic activity that Newman would have held to be appropriate to a university, albeit it may well be useful and entirely fitting for external organizations, or perhaps non-university-based research centers, to conduct predominantly this kind of work.

Liberal knowledge is relatively comprehensive. Someone who has liberal knowledge is not simply a narrow specialist. Here Newman is not putting forward the absurd proposition that educated scientists must have a complete grasp of the whole of science; but he is emphasizing the dangerous blinkering that intellectual specialization can impose. It is not uncommon, for example, to hear scientists in the physical sciences suggest that the work of those outside the physical sciences, including philosophy, is not evidence-based. Perhaps this is true of certain debased philosophizing—as it would be of debased science, e.g., astrology—but as a claim about the intellectual discipline of philosophy per se, it is manifest nonsense. Philosophical theories are typically subjected to rigorous testing by recourse to empirical facts, logical consistency, and so on.

Newman held that universities ought to teach the main areas of human knowledge, though not necessarily all areas of knowledge. He argued that intellectual vacuums tend to be filled by other existing disciplines. “For instance, I suppose, if ethics were sent into banishment, its territory would disappear, under a treaty of partition, as it may be called, between law, political economy and physiology” (Newman 1947, p. 65). Indeed, this process evidently took place in the twentieth century in relation to ethics when philosophers largely abandoned the study of the substantial ethical questions addressed from time immemorial, e.g., how to lead one’s life, how to structure a polity, in favor of formalist work in ‘meta-ethics’. Naturally, the point here is not to argue for practical ethics and *against* ethical theory; both kinds of study are required.

An important question which arises here is: Are there some academic disciplines that ought to be taught and/or researched in any contemporary institution wanting to be thought of as a university? Arguably there are and these should include philosophy, history and mathematics; mathematics because it is the language of science, history because understanding of the present presupposes understanding of the past, and philosophy because it is the academic discipline which integrates the understandings offered by other disciplines and derives an overall and synoptic conception of reality.

The need for certain specific disciplines to be taught in each and every individual university is consistent with a focus on the purpose of the system as a whole and the attendant possibility of a degree of specialization at individual universities, e.g., universities of technology. The point to be stressed here is one that Newman emphasized: there is a need for ‘liberal knowledge’ even—perhaps especially—among those specializing in science and technology for the reasons articulated by Newman (see above and below). The need to engender a capacity to understand the ethical issues that have proliferated as a consequence of new and emerging science and technology are merely the latest dramatic illustration of this point.

A related question pertains to the content of specific disciplines to be taught and researched, and how this is to be determined. Of importance here is the epistemic significance of the content under consideration. Presumably philosophy ought to be taught and researched but what ought its content to be? Is it a matter of following the intellectual interests of ‘academic stars’? Is there a need to ensure the preservation of the philosophical ideas of the past and build upon them? What are the most epistemically significant contributions?

Further questions pertain to the appropriate teaching, learning and research practices and methodologies appropriate to the content of specific disciplines in, for example, a rapidly changing teaching and learning technological environment, e.g., so-called MOOCs (Massive open online courses).

Newman stressed the role of the university in the transmission of knowledge and understanding, and thereby the preservation of societies. Here there is a backward looking and a forward looking aspect. On the one hand, this implies that it is important to look backward and maintain subjects like history, including the history of philosophical thought, and literary studies. On the other hand, now looking forward, the universities have a key role in contemporary communities facing challenges such as climate change, poverty and terrorism.

Moreover, once the content or, if you like, the questions, have been settled for each ‘intellectual generation’ by whatever means, there is the matter of the intellectual quality of the answers. Contrary to the impression that one might gain from the fixation with academic league tables, academic excellence is not in itself a status—high, medium or low. Rather it is an intellectual achievement consisting of the acquisition of understanding of epistemically significant questions and/or socially/economically problems based on adherence to high scholarly standards. As with any important human achievement, a reasonable measure of status should be accorded to academic excellence in this sense; but the two notions should not be confused, and status should not be privileged at the expense of excellence—indeed, the reverse should obtain.

Here it is important to recall the teleological normative theory of social institutions elaborated above, and to stress the essentially collective character of intellectual, including scientific, achievements, both at the individual and the institutional level; something which ranking systems, the Nobel Prize and the like ignore or downplay.

Consider in this connection the following points recently made in an opinion piece by Sean Carroll regarding the Nobel Prize:

Like many ideas in physics, the Higgs mechanism came together with contributions from many different people, including renowned physicists like Philip Anderson, Robert Brout, Gerald Guralnik, Carl Richard Hagen and Tom Kibble. But only Mr. Englert and Mr. Higgs are sharing the Nobel…Why not give it to them all? Because there is a tradition that the science prizes are given to individuals, not to collaborations — and to no more than three individuals in any one year. That tradition needs to end. Science has always been an intensely collaborative pursuit, and prizes to individuals are rarely able to capture the full nuance of the historical reality. In the modern era, when communication between scientists anywhere on earth is instantaneous and effortless, collaborations are growing larger and more central to the scientific enterprise… I’m not suggesting that 6,000 people should each receive a Nobel Prize. Rather, this year’s award highlights the basic flaw with science awards themselves: there is no way a prize like the Nobel can accurately capture the intricate history of any individual discovery. At the very least, in the future the prize committee should be allowed to consider institutions and collaborations as well as individuals. Doing so would not only give recognition to the many people who deserve credit for discoveries like the Higgs, but it would also reflect to the world the nature of modern scientific investigation. (Carroll 2013)

Newman also held that the professions are a proper part of the knowledge that universities ought to teach. It remains an open question as to which professions ought to be taught in any given university.

Newman is committed to the view that liberal knowledge is a good in itself, and that it needs to be taught in order to be acquired. In this essentially epistemic context he stressed the intellectual virtues—as opposed to the moral virtues. The intellectual virtues include the capacity to think logically, to communicate clearly, the habit of reflection and of intellectual curiosity about one’s environment, the need for independence of judgment, especially in the face of external pressure and fashion, and the motivation to seek objective truth.

Here it must also be stressed that intellectual empowerment of the sort that Newman advocates is of enormous benefit not only to the individuals who gain it, but to the wider society, including the economic system. Liberal knowledge is not only a good in itself, it also has great utility for society at large.

Finally, Newman’s emphasis on independence of judgment based on reasoning and driven by a desire for objective truth, remains of fundamental importance in the contemporary world, dominated as it is, by powerful interests, organizations and associated ideologies, and increasingly by religious fundamentalism of various kinds. As the late Edward Said put it, academics have a duty to: “speak the truth to power” (Said 1994). But equally, as Newman said:

Knowledge is capable of being its own end…What the worth of such an acquirement (knowledge) is, compared with other objects that we seek – wealth or power or honor or the conveniences and comforts of life, I do not here profess to discuss; but I would maintain that (knowledge) is an object, in its own nature so really and undeniably good, as to be the compensation of a great deal of thought in the compassing, and a great deal of trouble in the attaining. (Newman 1947, p. 91).

Let us now turn briefly to some criticisms of Newman’s normative conception of a university; criticism that are salient in the light of our concerns in this paper. As is often pointed out, Newman believed that research has no place within a university. In this matter Newman is surely wrong since many students need to be inducted into research activities and this can only be achieved in an environment where teachers are also researchers. However, it is important to see that the idea of academics both teaching and researching is not alien to Newman’s fundamental conception. For his conception of a university education involves the *transmission* of an intellectual culture through teaching, and the presentation of that culture’s characteristic outlooks and virtues. But these depend upon students viewing their teachers as people who are not merely *handing on* some lumps of established fact but who are participants in the intellectual debate, exploration and (to use a term of Michael Oakeshott’s) conversation of our culture and our species. Of course, the term “research” is capable of multiple construal, but if we use it so as to encompass serious participation in the “conversation”, then it must have an important place in the university.

Newman was correct in pointing out some of the problems of the combination of research and teaching functions viz. inadequate time to research, inability of some teachers to produce original research and of some researchers to teach effectively. It may be that graduate schools are part of the answer to these sorts of problems. There are many complications that would need to be addressed in a fuller treatment of these issues, but what I am chiefly concerned to do here is to point out that a research function for universities, far from being inconsistent with Newman’s vision of higher education actually complements it.

It is often objected that Newman in stressing liberal knowledge for its own sake fails to understand that universities must be useful to the wider society. For example, Derek Bok, former president of Harvard University, makes this sort of claim (Bok 1990, p. 70). This objection pertains both to social and economic utility.

For reasons of space let us simply look at economic utility. Newman is keen to sing the praises of what he calls the philosophical cast of mind as something absolutely central to university education, but he does not see this outlook or set of attitudes, as restricted to any particular subject matter. As we have already seen he is perfectly clear that professional disciplines have their place in the university, as they had from its medieval beginnings: what he is concerned to stress is that in addition to professional training one must aim at the intellectual virtues; indeed, the inculcation of the intellectual virtues ought to be part and parcel of professional ‘training’ (or, rather, education).

One central value of higher education is its power to enlarge the understanding and imagination, to produce a perspective on the particular facts and skills which are learned. This includes an understanding of the limits and complexities of present understanding; in the professions, as elsewhere, it is important to know how little you know even when you are on top of your subject. But none of this involves any essential hostility to professional education, including the kind of professional education typically offered in universities of technology, such as architecture and engineering.

Other central values of higher education stressed by Newman are those intellectual virtues that might be termed rational capacities. Indeed these intellectual virtues are precisely what employers are now beginning to realize are necessary for the economic system. The capacity to think logically, to communicate effectively, to focus on the key points in any issue, to absorb new knowledge speedily; these are in fact the necessary ingredients for the bringing into existence of a competitive modern economy. But Newman is right to stress that these virtues or rational capacities are hard won and only reliably acquired by large numbers of people in an institutional setting which has the appropriate intellectual traditions and which has teachers who have spent long years absorbing these traditions. These traditions and teachers cannot simply be wished into existence by setting up a committee and drawing up a report in which these rational capacities are pronounced desirable.

## Institutional Purpose and Universities of Technology

In the light of the above discussions, let me now make a number of key points that are germane to the institutional purposes (normatively speaking) of universities of technology in particular.

Firstly, scientific research is essentially collaborative in character; the collaboration in question being both at the individual and the institutional (including transnational) levels.

Secondly, important scientific findings and their technological applications are ones that are globally relevant, if not directly then indirectly; the cure for cancer, for example, is obviously directly relevant to the populations of all nation-states but, perhaps less obviously, vaccines for poverty-related infectious diseases are indirectly relevant to the populations of ‘rich’ countries, given global interdependence.

Thirdly, there is a need to identify and pursue research that is both epistemically significant as well as being socially and economically beneficial. Moreover, the relationship between these ends is a complex one; simplistic instrumentalist conceptions are not to the point (more of this below).

If these points are well-made, then the most appropriate normative institutional model might well be a model not simply of a single organisation considered on its own, e.g., Delft University of Technology, but rather of a higher education system (say, the Dutch higher education system), indeed, of structured sets of such systems (e.g., the IDEA league).^1^ The appropriateness of this model is evident when—by the lights of our normative teleological model of social institutions—one considers the following: (1) the nature of the collective good in question (roughly speaking, scientific understanding); (2) the manner of its ‘production’ i.e., by the joint activity of globally networked scientists; and (3) the aggregate epistemic, economic and social needs that scientific understanding and its technological applications serves, i.e., those of multiple intersecting global communities.

Notice that it is consistent with this model that any given university have a set of segmented but overlapping ‘communities’ which it serves. For example, a university in the Netherlands might principally provide undergraduate teaching services to members of the Netherlands population (and be funded by the Netherlands government principally to do so), but also undertake certain research jointly with other overseas universities which it made available to the international community.

Given this model of universities of technology and systems thereof, what is to be made of hybridisation: the threefold process of massification, marketization and bureaucratization described in the opening section of this paper?

Bureaucratization is a process in need of resistance. Certainly large modern organisations—in some cases, arguably, too large, e.g., banks that are too big to fail, multiversities that are too big to manage(?)—including universities, require an hierarchical administrative arm to provide services for academic staff and students, e.g., application processes, processing of grades, salary and scholarship payments, and general running of the university, e.g., maintenance. Moreover, there is a need to hold all personnel including, but not restricted to, academic staff, accountable in relation to their performance. However, bureaucratization is simply a means to an end; at least in universities it is not an end in itself. Yet the growth of administrative personnel relative to academic staff, the proliferation of administrative agencies, e.g., marketing agencies, academic accountability sections, research offices etc., and the constant restructuring of universities departments, schools, faculties and so on, are typically not themselves subject to detailed independent scrutiny and justification. Indeed, arguably in many universities bureaucratization has become, in effect, an end-in-itself for administrative managers, in particular. Moreover, as mentioned above, there has been a shift in governance structures from collegial to bureaucratic/hierarchical. This is problematic. For given that the fundamental institutional purposes of universities are academic, e.g., the disinterested pursuit of epistemically significant knowledge for its own sake, and, as such, principally dependent on the research and teaching abilities of academics, it is extremely doubtful that a top-down hierarchical structure is preferable to an essentially bottom-up collegial one.

A particular problem here is the tendency of bureaucratic processes of performance assessment to fixate on what is quantifiable and, therefore, able to be adjudicated by bureaucrats, e.g., numbers of publications and citations, numbers of students, research dollars generated, at the expense of what is, in the end, actually required but is inherently unquantifiable, namely, judgments of academic quality and epistemic significance made by relevantly knowledgeable academics.

Arguably, massification of universities in general, and universities of technology in particular, is inevitable given the existing and emerging needs of modern economies and, notably, their dependence on technology. Accordingly, higher education systems, including universities of technology, have to manage the process in such a way as to allow for the systematic and orderly education of large numbers of students while ensuring that the essentially epistemic purposes of universities are realized, and the requisite scientific and scholarly values and standards are preserved and transmitted to future generations of scientists and scholars. This is, of course, easier said than done in the context of very significant under-resourcing of many, if not most, universities in the US, UK, Europe, Australasia and elsewhere. However, this is not the place to attempt to square this particular circle.

The marketization process in higher education and especially in universities of technology is, by the lights of our normative model, a double-edged sword. On the one hand, markets induce competition and as such usefully induce striving for excellence, innovation and the acquisition of new knowledge. Moreover, student and research markets served by universities, including ones served as a consequence of government intervention in the form of government funding or by other less direct means, are often ones that on social or economic grounds ought to be served, e.g., university-based, government funded research on poverty related diseases. On the other hand, competitive markets tend to promote competition at the expense of cooperation, individual and institutional achievement and status at the expense of an overriding commitment to epistemic values, short term technological breakthroughs at the expense of long term fundamental research, economically—as opposed to socially—important research, powerful economic interests at the expense of less powerful but more pressing ones, and so on. Here I need to briefly note an important feature of businesses operating in a competitive market which creates special problems for certain kinds of social institutions and, specifically, for universities, namely profit maximisation.

Market-based organizations have profit maximisation as a collective end. The existence of profit maximization adds a complication in the case of market-based organizations that is not present in the case of other social institutions.

All social institutions have what might be referred to as *constitutive* collective ends and *constitutive* collective goods. Thus car manufacturers have the production of cars as a constitutive collective end and transport as a constitutive collective good; universities have the generation of scientific understanding as a constitutive collective end and as a constitutive collective good. However, market-based institutions have an additional non-constitutive end, namely, profit maximisation.

It is in the collective self-interest of managers and owners of businesses, e.g., shareholders, to maximise revenue and profit and minimise costs. So profit maximization is a collective end to which the constitutive collective end is a means, e.g., cars are produced and sold for profit, research is produced and sold for profit by private sector research firms.

Accordingly, there are now two potentially competing collective ends, namely, collective goods and profit maximization. However, as is well known, a solution of sorts has been offered to this problem, namely, the so-called invisible hand. The claim is that the single-minded and self-interested pursuit of profit will, as a matter of contingent fact, maximize collective benefits (on some construal of collective benefits, e.g., utilitarianism). Relativized to our account of social institutions, the claim is that the pursuit of the collective end of profit maximization as an end will, as a matter of contingent fact, realize the collective good definitive of the social institution in question.

Unfortunately, the empirical claim upon which the efficacy of the invisible hand is predicated is contestable and, in some cases, evidently false. In particular, the claim is false in the case of some social institutions which have a constitutive collective end that is identical, at least in part, with their defining collective good, notably media institutions but also universities. For in these cases there is a tendency for the collective good to be discounted, or even regarded simply as a means to profit maximization or related economic ends—rather than the other way around. Evidently this is to some extent inevitable, given the constitutive collective end, e.g., the acquisition and dissemination of news or of research-based knowledge, is regarded by owners and managers alike as a means to profit maximization, and yet the constitutive collective end is itself the collective good (Miller 2010). At any rate, as Resnik and others have argued, in universities of technology in particular, student recruitment and courses offered, and research in science and technology have become heavily influenced by market-based considerations and, specifically, by considerations of profit, revenue generation and the like. Arguably research in science and technology in universities of technology has been skewed (directly or indirectly) by market-based considerations at the expense of the consideration of epistemic significance. According to Resnik, “The vast majority of scientists depend on government or industry contracts or grants to support their research and study problems that are of interest to those who fund research” (Resnik 2007, p. 79). And there is this further related point. Market based institutions, for instance in the information and communication technology sector, have in some instances (e.g., Facebook, Google, Amazon) become so wealthy and powerful that they are able to attract large numbers of leading researchers from the universities, and the universities of technology in particular, that there is a risk that research in these areas will largely—and now *directly*—be driven by market-based considerations rather than by considerations of the collective good.

Thus far I have assumed, consistent with Newman’s conception, that the constitutive collective end and constitutive good of universities is an epistemic one; specifically, in the case of universities of technology, epistemically significant, scientific understanding pursued disinterestedly and for its own sake as well as for its economic and social benefits. I note in passing that in many areas of science, e.g., the biological sciences, the distinction between science and technology is far from clear-cut and that the understanding in question is as much about how to engineer or make things as it is about how to understand pre-existing things; and that Newman’s notion of liberal knowledge is perhaps in need of renovation in this regard. However, as already mentioned, and notwithstanding Newman’s insights outlined above, the claim that the fundamental institutional purpose (constitutive collective end and good) of universities and systems of universities is an epistemic one has been disputed. I do not have the space here to defend this assumption—other than by invoking Newman—but in closing I do want to point out some important untoward potential consequences of its rejection.

Firstly, it leaves the door open for utilitarians, instrumentalists and ideologues of all persuasions to colonise or, perhaps, marginalize the university in the service of their own organisational purposes, be they economic, social or political and, thereby, jeopardises the wherewithal for sustained and independent intellectual understanding and critique of these very colonising organisations themselves, be they market-based, governmental or other.

Secondly, it clears the way for the de facto removal of the primary institutional centre in the modern world for the disinterested pursuit of epistemically significant knowledge for its own sake, including scientific understanding, and, thereby, undercuts the ages old human epistemic project itself.

One indicator that this latter project might already be in trouble, notwithstanding the exponential increase in instrumentally–based knowledge over the last few decades, is the marginalising in both strategic and specific policy oriented discussions in universities of the very notion of epistemically significant knowledge (as opposed to the notion of instrumentally important knowledge).

A second indicator is the relative decline of the discipline of philosophy within universities. For what I have referred to as the human epistemic project ultimately seeks a synoptic and systematic overview of reality in all its main aspects, physical and psychological, microscopic and macroscopic, descriptive and normative, and, therefore, requires not only the understanding of each of the (so to speak) fragments of that reality—understandings provided by particular sciences—but also a coherent account of the relationship between those fragments, e.g., the relationship between brain states and consciousness, the relation between the descriptive and the normative—an account best provided by philosophy.

## Footnotes

1 IDEA league is a European network of technical universities comprised of Delft University of Technology (Netherlands), ETH Zürich (Switzerland), RWTH Aachen University (Germany), Chalmers University of Technology (Sweden), and the University Politecnico di Milano (Italy).

## References

[CR1] Barnett R (1990). Idea of higher education.

[CR2] Barnett R (2013). Imagining the university.

[CR3] Bok D (1990). Universities and the future of America.

[CR4] Bourke P (1986). Quality measures in universities.

[CR5] Brennan J, Tahla S (2000). Managing quality in higher education.

[CR6] Carroll, S. (2013). No physicist is an island. *International Herald Tribune* Oct. 8 Edition.

[CR7] Coady CAJ, Miller Seumas (1993). Australian higher education and the relevance of Newman. Australian Universities Review.

[CR8] Jaspers K (1960). The idea of the university.

[CR9] Miller S (1992). Joint action. Philosophical Papers.

[CR10] Miller S, Coady CAJ (2000). Academic autonomy. Why universities matter.

[CR11] Miller S (2001). Social action: A teleological account.

[CR12] Miller S (2010). The moral foundations of social institutions.

[CR13] Miller S (2015). Joint epistemic action and collective moral responsibility. Social Epistemology.

[CR14] Miller S (2016). Assertions, joint epistemic actions and social practices. Synthese.

[CR15] Miller S (2018). Dual use science and technology, ethics and weapons of mass destruction.

[CR16] Newman JH (1947). Idea of a university.

[CR17] Pelikan J (1992). The idea of the university: A reexamination.

[CR18] Resnik DB (2007). The price of truth: How money affects the norms of science.

[CR19] Said E (1994). “Lecture V—Speaking truth to power” in his Representations of the intellectual: The 1993 Reith lectures.

[CR20] Slaughter S, Leslie L (1997). Academic capitalism: Politics, policies and the entrepreneurial university.

